# Genetic diversity and genetic relationships of *japonica* rice varieties in Northeast Asia based on SSR markers

**DOI:** 10.1080/13102818.2014.908019

**Published:** 2014-06-16

**Authors:** Jingguo Wang, Tingbo Jiang, Detang Zou, Hongwei Zhao, Qiang Li, Hualong Liu, Changjun Zhou

**Affiliations:** ^a^ State Key Laboratory of Tree Genetics and Breeding, Northeast Forestry University, Heilongjiang, P.R. China; ^b^ The Rice Research Institute, Northeast Agricultural University, Heilongjiang, P.R. China; ^c^ Heilongjiang Province Economic Research Institute of State Farm, Heilongjiang, P.R. China; ^d^ Heilongjiang Agriculture Academic Sciences, Daqing Branch, Heilongjiang, P.R. China

**Keywords:** *japonica* rice, genetic diversity, simple sequence repeat (SSR), Northeast Asia

## Abstract

Genetic diversity and the relationship among nine *japonica* rice groups consisting of 288 landraces and varieties in different geographical origins of Northeast Asia (China, Japan, Korea, Democratic People's Republic of Korea) and the Russian Far East district of the Russian Federation were evaluated with 154 simple sequence repeat (SSR) markers. A total of 823 alleles were detected. The observed allele numbers (Na) per locus, Nei's gene diversity (He) and the polymorphism information content (PIC) ranged from 2 to 9, 0.061 to 0.869 and 0.060 to 0.856, with an average of 5.344, 0.624 and 0.586, respectively. Five SSR loci, RM1350, RM1369, RM257, RM336 and RM1374, provided the highest PIC values and are potential for exploring the genetic diversity of rice cultivars in Northeast Asia. Molecular variance analysis showed that a significant difference existed both among groups (91.6%) and within each group (8.4%). The low genetic variation within each group indicated that the gene pool is narrow and alien genetic variation should be introduced into the rice breeding program in Northeast Asia. Based on the He and PIC values, the nine groups were ranked in a descending order: Heilongjiang landraces, Jilin landraces, Japanese improved varieties, Heilongjiang improved varieties, Russian Far East district of the Russian Federation improved varieties, Liaoning improved varieties, Jilin improved varieties, Korean improved varieties and Democratic People's Republic of Korea improved varieties. The nine groups were further divided into three subgroups and the 288 varieties into five clusters. This study provided information for parent selection in order to broaden the gene pool of the *japonica* rice germplasm in Northeast Asia.

## Introduction

Rice (*Oryza sativa* L.) is one of the most important crops in the world. Northeast Asia includes the areas of Northeast China (Heilongjiang, Jilin and Liaoning Provinces), Japan, Korea, Democratic People's Republic of Korea and Mongolia, along with the area of the Russian Far East district of the Russian Federation.[1] In 2010, in this area (excluding Mongolia) the planted rice reached 7.617 million hectares, accounting for 4.96% of the world records, and the total production reached 48.09 million tons, accounting for 7.23% of the world produce.[2]

The study of genetic diversity is of great significance to the effective conservation and optimal use of the vast gene resources. In recent years, simple sequence repeats (SSRs), with the advantages of allele specificity and co-dominance, have been extensively used to assess genetic diversity and the relationships of subspecies among rice cultivars.[3–6]

Few research studies on the genetic diversity and genetic relationships of different *japonica* rice cultivars and landraces from different geographical regions in Northeast Asia have been reported until now. In this study, 154 SSR markers on 12 rice chromosomes were randomly selected to investigate the genetic diversity and the genetic relationships among the 288 accessions (included landraces and improved varieties) in different geographical areas (Heilongjiang, Jilin and Liaoning Provinces of Northeast China, Japan, Korea, Democratic People's Republic of Korea, along with the Russian Far East district of the Russian Federation). This research will acknowledge the present situation, characteristics, developing trend and improvement emphasis of the germplasm resources in Northeast Asia, and provide references for protecting the genetic diversity, broadening the genetic pool and the effective utilization of germplasm resources.

## Materials and methods

### Materials

In this study, 288 rice accessions (landraces and improved varieties) were collected from the Crop Science Research Institute, Chinese Academy of Agricultural Sciences, Liaoning Academy of Agricultural Sciences, Heilongjiang Academy of Agricultural Sciences and Northeast Agricultural University. Based on their geographical distribution and variety types, these accessions were divided into nine groups: Heilongjiang landraces (HL), Jilin landraces (JL), Heilongjiang improved varieties (HIV), Jilin improved varieties (JLIV), Liaoning improved varieties (LIV), Japanese improved varieties (JIV), Korean improved varieties (KIV), Democratic People's Republic of Korea improved varieties (DPRKIV) and the Russian Far East district of the Russian Federation improved varieties (RFERIV). Each group consisted of 53, 51, 25, 26, 29, 60, 28, 9 and 7 accessions, respectively (Table S1 in the Online Supplemental Appendix).

### Total genomic DNA extraction

Total genomic DNA was extracted and purified from the young leaves by a modified CTAB method described by Edwards et al. [7]. The DNA quality and concentration were examined in a 0.8% agarose gel in 1× TBE buffer (0.09 mol/L Tris–borate and 0.5 mol/L EDTA) at 80 V for 90 min and stained with ethidium bromide.

### Primers and polymorphic examination

Based on the SSR-contained sequence information at http//www.gramene.org, 600 primer pairs on rice chromosomes were designed and synthesized by the Sangon Biotech Co. Ltd. (Shanghai). Twelve genotypes from the nine groups (Laotoudao 1, Baidadu, Kendao 12, Xiaobaijingzihuadianbai, Jijing 61, Danjing 8, Shennong 91, Fuchihikari, Kuiku 131, Woonbongbyeo, Jinbooolbyeo, and Pyeongyang 15) were selected for testing the polymorphism of the primers. Finally, 154 out of 600 primers with higher amplification rate and distinct polymorphism were selected for the genetic diversity study (Table S2 in the Online Supplemental Appendix).

### PCR amplification

PCR reaction was conducted in 20 μL volumes mixed with 2 μL of genomic DNA (25 ng/μL), 1.5 μL of MgCl_2_ (25 mmol/L), 0.3 μL of dNTP mixtures (10 mmol/L), 2 μL of 10 × PCR buffer, 2 μL of SSR primer (2 μmol/L), 0.2 μL of Taq polymerase (10 U/μL) and 12 μL of ddH_2_O. The amplification profiles were 94 ºC for 2 min, followed by 35 cycles of 94 ºC for 30 s, 47 ºC for 30 s, 72 ºC for 30 s, and then extended at 72 ºC for 5 min.

### Products separation and detection

PCR products were mixed with loading buffer (2.5 mg/mL bromophenol blue, 2.5 mg/mL diphenylamine blue, 10 mmol/L EDTA, 95% formamide) and denatured at 94 ºC for 5 min, and then put on ice for 5 min. The denatured PCR products were separated in a 6% denaturing polyacrylamide gel and directly detected by silver straining.[8]

### Data collection and analysis

Clearly detectable polymorphic bands were scored for the analysis. Each amplified polymorphic band was assigned as one allele for each SSR locus. Band presence or absence was scored as 1 or 0 within each accession. POPGENE 1.32 [9] was used to calculate the genetic identity, genetic distance, coefficient of differentiation (Fst) and gene flow (Nm) between the nine groups [10,11]. Na, He and PIC [12] were calculated with the program PowerMaker 3.25.[13] The data were analysed with the qualitative routine to generate Jaccard's similarity coefficients. Similarity coefficients were used to generate dendrograms, using the UPGMA (unweighted pair group method with arithmetic average) and the SHAN (sequential, hierarchical and nested clustering) methods of the NTSYS-pc 2.1 software.[14] The molecular variances within and among groups were calculated using analysis of molecular variance (AMOVA) under GenAlEx6.2.[15]

## Results and discussion

### SSR polymorphism

A total of 823 allelic variations were detected using the 154 SSR primers (Table S2 in the Online Supplemental Appendix). Na ranged from 2 (RM272, RM292, RM345, RM346 and RM1210 on chromosomes 1, 6 and 7) to 9 (RM1347, RM1350, RM1369, RM336, RM1306, RM1353, RM257 and RM1374 on chromosomes 2, 3, 6, 7, 9 and 10), with an average of 5.34. He ranged from 0.061 to 0.869, with an average of 0.624. PIC ranged from 0.060 to 0.856, with an average of 0.586. Shannon's information index (*I*) varied from 0.042 to 2.070, with an average of 1.144. Five loci, RM1350, RM1369, RM257, RM336 and RM1374, on chromosomes 3, 6, 9, 7 and 10 ranked the top five on He, PIC and Na, indicating that these primers have potential to explore the genetic diversity for other rice germplasm in Northeast Asia. RM207, RM264, RM1306, RM501 and RM1379 showed the sixth to tenth largest PIC value, and RM207, RM264, RM1306, RM501 and RM1379 presented the sixth to the tenth largest He, while RM1306, RM1353, RM1347, RM207 and RM264 had the sixth to the tenth largest Na.

It is essential to understand genetic diversity for the effective conservation and utilization of rice germplasm. Previous studies on the genetic diversity of natural rice populations of improved varieties and landraces have been reported. Zhao et al. [16] used 29 SSR primers to analyse the genetic diversity of 150 accessions of cultivated rice from Korea, China and Japan. The Na obtained was 12.9, the mean PIC was 0.6683 and the mean He was 0.7001. Giarrocco et al. [17] surveyed 69 accessions with 26 SSR markers to reveal the genomic relationship among cultivars in Argentina. The Na obtained was 8.4, and the mean PIC was 0.69. Thomson et al. [18] characterized 330 rice accessions, using 30 microsatellite markers. The Na obtained was 13, and the mean PIC was 0.66. Shu et al. [19] studied the genetic diversity of 313 improved *japonica* varieties from 20 countries with 34 SSR primers and obtained an Na of 12.9 and a mean He of 2.8471. Obviously, the three parameters (Na, He and PIC) in this study were smaller than those in the aforementioned studies, indicating that the genetic diversity of these selected rice accessions in Northeast Asia was relatively low.

### Genetic diversity among different groups

Molecular variance analysis showed (Table 1) that a significant difference existed among groups and within each group (*P* < 0.01). The variation within each group accounted for 8.40% and that among the groups for 91.60%. Therefore, it is necessary to do further analysis of the genetic diversity among different groups.

**Table 1.** AMOVA among different groups and within one group.

Source	df^a^	SS^b^	MS^c^	Est. Var.^d^	Var.^e^%	*P*
Among Pops	8	5471.798	683.975	16.417	8.40	<0.01
Within Pops	279	49,920.327	178.926	178.926	91.60	<0.01
Total	287	55,392.125		195.343	100.00	

^a^df: degrees of freedom; ^b^SS: sum of square; ^c^MS: mean square; ^d^Est. var.: estimated variance; ^e^%Var.: percentage of total variance.

Based on the He and PIC value (Table 2), the nine groups were ranked in a descending order as follows: HL, JL, JIV, HIV, RFERIV, LIV, JLIV, KIV, DPRKIV. The mean He and PIC of landraces (HL and JL) was 0.624 and 0.577, and that of the improved varieties was 0.537 and 0.488, respectively. Apparently, the genetic diversity in landraces (HL and JL) was much larger than that of the improved varieties, indicating that landraces have a wider range of genetic variation than the selected varieties.

**Table 2.** Genetic diversity of groups.

Group	Na^a^	He^b^	PIC^c^
HL	4.435	0.641	0.596
HIV	4.013	0.570	0.527
JL	3.994	0.606	0.559
JLIV	3.377	0.522	0.476
LIV	3.630	0.528	0.483
JIV	4.506	0.572	0.530
KIV	3.481	0.513	0.469
DPRKIV	2.532	0.501	0.441
RFERIV	2.708	0.551	0.488

^a^Na: observed number of alleles; ^b^He: Nei's genetic diversity index; ^c^PIC: polymorphism information content.

Na, He and PIC are widely used to quantify the level of the genetic diversity of plant species. However, the explanation of these parameters is conflicting. For example, Xu et al. [20] found that the genetic diversity of *indica* rice cultivars was higher than that of *japonica* cultivars, although the number of accessions and Na of *indica* accessions were less than those of *japonica* cultivars. Sun et al. [21] also reported that although the average gene diversity of the South Asian common wild rice was higher than that of the Southeast Asian common wild rice, its percentage of polymorphism per loci, Na and number of genotypes all were smaller. The same parametric relationship emerged in this study. JIV had a larger number of accessions and Na than those of HL, but He and PIC were lower than those of HL. Actually, HL was a landrace with less improvement, and therefore it retains more genetic variation. In contrast, JIV has undergone longer breeding improvement with interspecific hybridization so it was found to have a narrow genetic background. This indicates that He and PIC values were more easily affected by the variety improvement status.

Previous studies have compared the genetic diversity among improved varieties, landraces and common wild rice from different countries and regions. The results of Shu et al. [19] indicated that the genetic similarity (GS) of the varieties from the north of China was higher compared to that of varieties from Korea, DPRK and Japan. Zhao et al. [16] found that the genetic diversity of the Korean and Chinese cultivars was higher than that of the Japanese cultivars. In contrast, the nine groups in this study were ranked in a descending order based on the He and PIC values, which indicated that HL and JL had the highest genetic diversity, and JIV showed higher genetic diversity than KIV.

### Genetic relationships among different groups

Compared with the genetic identity and genetic distance (Table 3) among different groups, it can be found that JIV had the highest genetic identity (0.921, 0.922 and 0.923) but the smallest genetic distance (0.082, 0.082 and 0.080) with HIV, JLIV and LIV. Except for HL, the other seven groups had the smallest genetic identity and the largest genetic distance with RFERIV.

**Table 3.** Genetic distance between groups.

	HL	HIV	JL	JLIV	LIV	JIV	KIV	DPRKIV
HIV	0.159							
JL	0.138	0.138						
JLIV	0.207	0.126	0.170					
LIV	0.210	0.164	0.202	0.090				
JIV	0.151	0.082	0.138	0.082	0.080			
KIV	0.239	0.171	0.270	0.144	0.161	0.124		
DPRKIV	0.238	0.183	0.230	0.123	0.131	0.126	0.161	
RFERIV	0.187	0.284	0.293	0.374	0.365	0.296	0.395	0.398

A comparison was made on the Fst and Nm in order to elucidate the differences of the genetic distance among the nine groups (Table 4). The results showed that Fst among the groups ranged from 0.036 to 0.164, with an average of 0.0811, indicating that there was 8.11% genetic variation among the groups and 91.89% variation within each group. This is in agreement with the results of the molecular variance analysis. Nm ranged from 1.272 to 6.678, with a mean value of 3.299, indicating that there was frequent genetic information exchange among the nine groups, i.e. rice varieties in the Northeast Asia region were frequently introduced and exchanged.

**Table 4.** Coefficient of differentiation (Fst, below diagonal) and gene flow (Nm, above diagonal) between groups.

	HL	HIV	JL	JLIV	LIV	JIV	KIV	DPRKIV	RFERIV
HL	****	4.136	5.338	2.826	2.991	4.449	2.442	2.335	3.274
HIV	0.057	****	4.458	3.902	3.223	6.678	2.884	2.619	2.072
JL	0.045	0.053	****	3.177	2.908	4.534	2.146	2.266	2.091
JLIV	0.081	0.060	0.073	****	5.112	5.890	3.060	3.414	1.491
LIV	0.077	0.072	0.079	0.047	****	6.446	2.902	3.289	1.594
JIV	0.053	0.036	0.052	0.041	0.037	****	3.961	3.528	2.003
KIV	0.093	0.080	0.104	0.076	0.079	0.059	****	2.626	1.417
DPRKIV	0.097	0.087	0.099	0.068	0.071	0.066	0.087	****	1.272
RFERIV	0.071	0.108	0.107	0.144	0.136	0.111	0.150	0.164	****

Similarly, the results in Table 4 showed that JIV had the highest Nm (6.678, 6.446, and 5.890) and the lowest Fst (0.036, 0.037 and 0.041) with HIV, LIV and JLIV, respectively. Except for HL, the other seven groups had the smallest Nm and the largest Fst with RFERIV. It can be concluded that JIV had the most frequent genetic information exchange with HIV, JLIV and LIV, and especially had remarkable influence on HIV. RFERIV had the least exchange with other groups, followed by KIV and DPRKIV. Compared with other groups, RFERIV and HL had the smallest genetic distance and the most frequent genetic information exchange with each other.

The following facts can be concluded according to the Nm of one group vs. each of the other eight groups (Table 4): the largest Nm values are those of HL versus JL and vice versa; of RFERIV versus HL; of JIV versus HIV and of JLIV, LIV, KIV and DPRKIV versus JIV. The Nm of HL and RFERIV versus DPRKIV are the smallest, and except for this, the Nm of the other seven groups versus RFERIV are the smallest.

Different Nm values reflect the difference in genetic information exchange among different groups in Northeast Asia: the HL and JL groups are greatly influenced by each other; RFERIV is greatly influenced by HL; HIV, JLIV, LIV, KIV and DPRKIV bear the largest influence by JIV, but the least by RFERIV; JL and JIV bear the least influence by RFERIV; HL and RFERIV bear the least influence by DPRKIV.

The genetic background of the parents must be fully considered in order to expand their gene pool in breeding programmes. Zhao et al. [22] studied the genetic variation of *japonica* rice cultivars from Yunnan, China and Korea, using SSR markers. The results showed that there was a significant difference in genetic variation between Yunnan and Korea rice cultivars. They suggested that the Korean varieties can be used to expand ‘Yunnan varieties’ genetic base and improve rice quality. Based on the genetic relationship of the nine groups, it is suggested that (1) HL and JL can be used more frequently to improve HIV, JLIV and LIV; (2) unfavourable influence caused by similar genetic background must be fully considered when using JIV to improve HIV, JLIV and LIV, especially for HIV; (3) RFERIV can be used to broaden the genetic base of rice cultivars from other countries or regions in Northeast Asia.

### Cluster analysis

**Figure 1.**
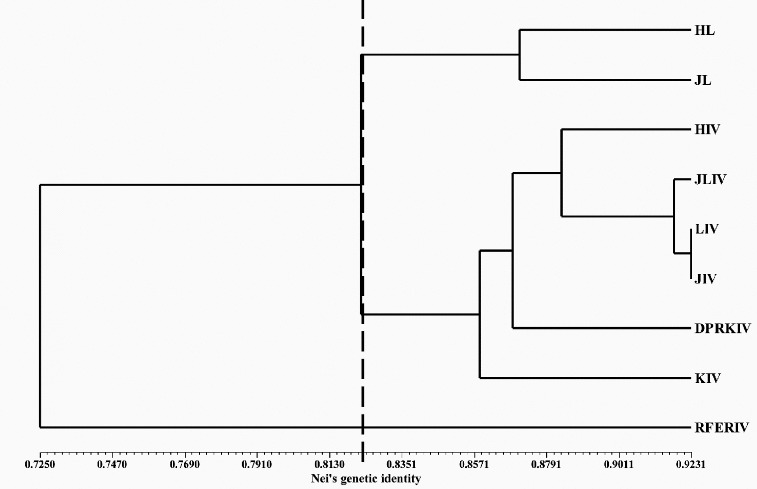
Dendrogram of nine *japanica* rice groups based on genetic identity, using the UPGMA and SHAN routine in the NTSYS 2.1 program. The vertical dotted line indicates the genetic identity value, 0.8212, dividing the nine groups into three clusters.

Based on genetic identity, the nine groups can be divided into three subgroups with a threshold of 0.8212. The first subgroup included HL and JL; the second subgroup included JLIV, HIV, LIV, JIV, DPRKIV and KIV; and the third subgroup included only RFERIV. In the second subgroup, HIV, JLIV and LIV were closely related to JIV, especially for LIV (Figure 1).

Based on the coefficient of genetic similarity, the 288 varieties can be divided into five clusters with a threshold of 0.6925 (Figure 2), consisting of 75, 34, 72, 35 and 72 varieties, respectively. Each cluster contained varieties from more than two groups (Table 5), revealing the complicated genetic information exchange among them. The second and fifth cluster (II and V) contained 98.1% HL, 88.0% JL and 100% RFERIV. The first, third and fourth cluster (I, III and IV) contained 84.3% HIV, 100% JLIV, 93.1% LIV, 81.7% JIV, 92.9% KIV and 88.9% DPRKIV. This composition further supported the above results: (1) the landrace varieties were clustered together, so were the improved varieties; (2) except for HL, the other seven groups had the largest genetic distance, the smallest Nm and the largest Fst with RFERIV; (3) RFERIV and HL had the smallest genetic distance and the most frequent genetic information exchange with each other.

**Table 5.** Accessions numbers of each cluster.

	HL	HIV	JL	JLIV	LIV	JIV	KIV	DPRKIV	RFERIV	Total
I		30	3	2	6	34				75
II	23	1	2			3	1	1	3	34
III	1			21	20	15	7	8		72
IV		12		3	1		19			35
V	29	8	20		2	8	1		4	72
Total	53	51	25	26	29	60	28	9	7	288

Figure 2. Dendrogram of 288 *japonica* rice accessions based on Nei's (9) coefficient of genetic similarity, using the UPGMA and SHAN routine in the NTSYS 2.1 program. Panels 1 to 4 display 72 accessions, respectively. The vertical dashed line indicates the coefficient of genetic similarity value, 0.6925, dividing the 288 varieties into five clusters.

**Figure:**
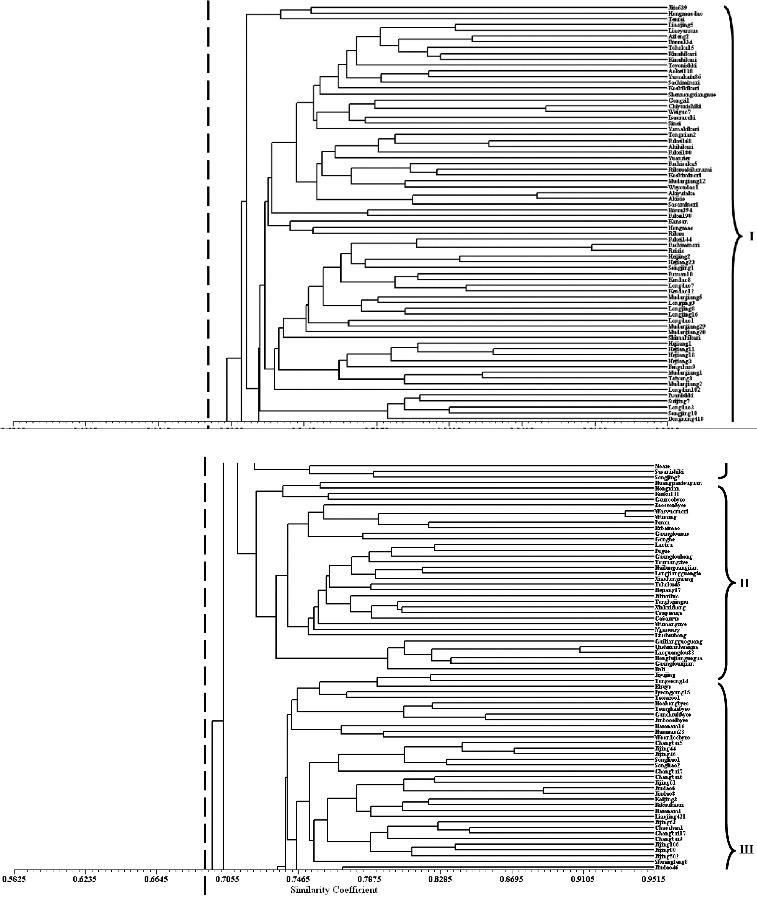

**Figure:**
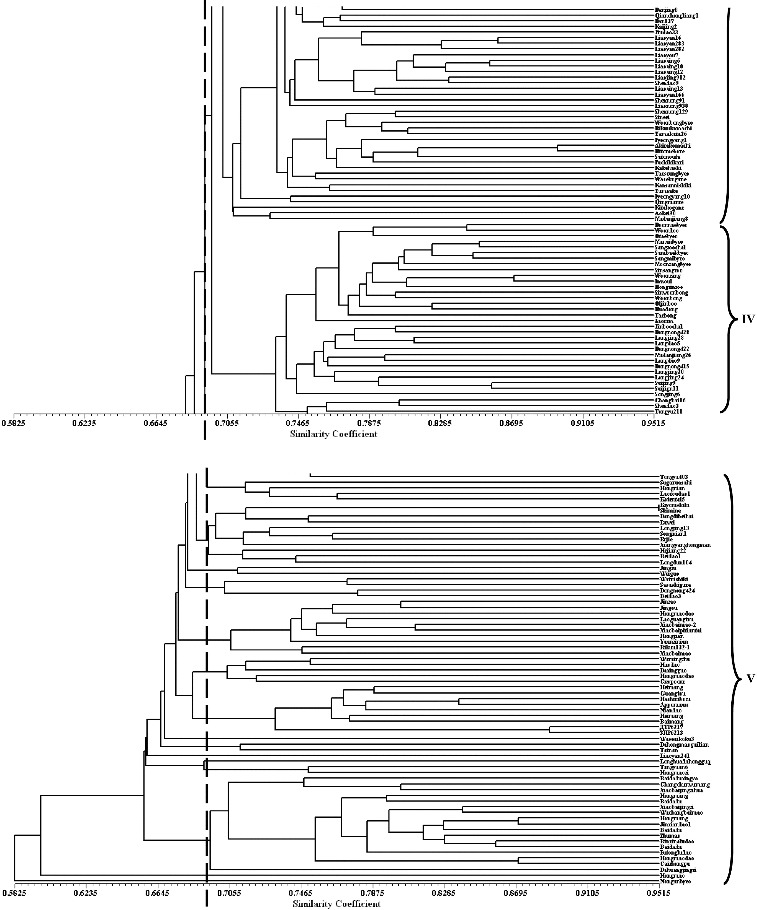


The first, second and fifth clusters contained 39 HIV and 45 JIV, accounted for 76.5% of HIV and 75.0% of JIV, respectively. The first and third clusters included 25 JLIV and 49 JIV, accounting for 88.5% of JLIV and 81.7% of JIV. The first, third and fifth clusters included 28 LIV and 57 JIV, accounting for 96.6% of LIV and 95.0% of JIV. This agreed with the above results that JIV had the most frequent genetic information exchange with HIV, JLIV and LIV, but it cannot explain that JIV had significant influence on HIV.

## Conclusions

In this study, the values of Na, He and PIC showed that the level of the genetic diversity of *japonica* rice cultivars in Northeast Asia was low, compared to previous studies. Molecular variance analysis showed that a significant difference existed both among and within groups. The nine groups were ranked in a descending order as: HL, JL, JIV, HIV, RFERIV, LIV, JIV, KIV, DPRKIV. The genetic relationships among different groups showed that genetic information exchange happens frequently.
